# A *Caenorhabditis elegans* model for ether lipid biosynthesis and function[S]

**DOI:** 10.1194/jlr.M064808

**Published:** 2016-02

**Authors:** Xun Shi, Pablo Tarazona, Trisha J. Brock, John Browse, Ivo Feussner, Jennifer L. Watts

**Affiliations:** School of Molecular Biosciences* Washington State University, Pullman, WA; Institute of Biological Chemistry,§ Washington State University, Pullman, WA; Department for Plant Biochemistry,† Albrecht-von-Haller Institute for Plant Sciences, Georg-August-University, Gottingen, Germany

**Keywords:** plasmalogens, lipidomics, phospholipids/phosphatidylethanolamine, fatty acid/desaturases, peroxisomes, *C. elegans*

## Abstract

Ether lipids are widespread in nature, and they are structurally and functionally important components of membranes. The roundworm, *Caenorhabditis elegans*, synthesizes numerous lipid species containing alkyl and alkenyl ether bonds. We isolated *C. elegans* strains carrying loss-of-function mutations in three genes encoding the proteins required for the initial three steps in the ether lipid biosynthetic pathway, FARD-1/FAR1, ACL-7/GNPAT, and ADS-1/AGPS. Analysis of the mutant strains show that they lack ether lipids, but possess the ability to alter their lipid composition in response to lack of ether lipids. We found that increases in de novo fatty acid synthesis and reduction of stearoyl- and palmitoyl-CoA desaturase activity, processes that are at least partially regulated transcriptionally, mediate the altered lipid composition in ether lipid-deficient mutants. Phenotypic analysis demonstrated the importance of ether lipids for optimal fertility, lifespan, survival at cold temperatures, and resistance to oxidative stress.*Caenorhabditis*

Cellular membranes are composed of a wide variety of lipid species, including phospholipids, cholesterol, and sphingolipids, which all play multiple roles in establishing a structural permeability barrier for cells and organelles, as well as provide an environment for proteins that participate in catalysis or function as channels and signaling molecules (1, 2). Most phospholipids consist of hydrophobic fatty acids attached to a hydrophilic phosphoglycerol head group through an ester bond. In contrast, ether-linked phospholipids are characterized by an ether bond connecting long chain fatty alcohols to the phosphoglycerol backbone (3, 4). Two types of glycerol ether bonds exist in nature, alkyl and alkenyl, in which the alkenyl ethers contain a *cis* double bond adjacent to the oxygen atom, and this class of lipids are also known as plasmalogens. Ether phospholipids ensure optimal cellular functions because their unique structures maintain membrane physical properties, including membrane fluidity, promotion of membrane fusion and contributing to the structure of lipid microdomains, and ability to serve as antioxidants (5). Ether lipids are also required for the proper function of integral membrane proteins and serve as precursors for lipid signaling molecules (6). Ether lipids are widespread in nature; however, the precise mechanisms in which ether lipids contribute to optimal membrane functions are still not clear.

The biosynthesis of ether lipids is vital to human health. Deficiencies in the peroxisomally localized ether lipid biosynthetic activities of glyceronephosphate *O*-acyltransferase **(**GNPAT) and alkylglycerone phosphate synthetase (AGPS) cause the human disease, rhizomelic chondrodysplasia punctata (RCDP). Babies born with these enzyme deficiencies have craniofacial abnormalities, shortened bones, cataracts, growth and mental deficiencies, and most do not survive beyond childhood (7). Recently, families carrying mutations in fatty acyl-CoA reductase (FAR)1 were identified, and these patients exhibit intellectual disability, cataracts, and other deficiencies similar to patients with RCDP (8). In addition, there is cumulative evidence for ether lipid deficiency in several other common disease states, including respiratory disease in premature infants and neurodegenerative disorders such as Alzheimer’s disease, Parkinson’s disease, and Neimann-Pick type C disease (6). In contrast, ether lipids are often elevated in human tumors, and the presence of ether lipids in cancer cells makes the tumors more aggressive (9). A recent study demonstrated the key role of the AGPS enzyme in promoting aggressive tumor growth (10). When AGPS was overexpressed, cellular ether lipids accumulated and tumor growth was promoted, while knockdown of AGPS impaired cancer cell survival, migration, and invasion (10).

Unlike the synthesis of conventional phospholipids, which occurs in the cytoplasm, the first steps of the synthesis of ether lipids occur in peroxisomes. Peroxisomes are vital organelles present in all eukaryotic cells that adjust their number in response to physiological conditions and metabolic needs (11). Peroxisomes play important roles in human physiology, as evidenced by the devastating diseases that result from peroxisomal biogenesis defects, as well as single enzyme peroxisomal deficiencies (7). Enzymes localized in peroxisomes perform both biosynthetic roles, such as ether lipid synthesis and bile acid synthesis, as well as essential catabolic functions, such as α- and β-oxidation of fatty acids, glyoxylate detoxification, and hydrogen peroxide metabolism (12). The association of peroxisomes with mitochondria and lipid droplets, along with their dual roles in carrying out anabolic and catabolic reactions, point to their importance in generating cellular signals required for normal physiological functions (13).

In a screen for *Caenorhabditis elegans* mutants with altered fatty acid composition, we identified strains carrying loss-of-function mutations in three genes encoding the proteins required for the initial three steps in the ether lipid biosynthetic pathway, FAR1, GNPAT, and AGPS. Our lipidomic study revealed many lipid species containing alkyl and alkenyl ether bonds in *C. elegans*. Analysis of these mutant strains shows that remarkable compensatory lipid composition changes occur in the absence of ether lipids. We found that increases in de novo fatty acid synthesis and reduction of stearoyl- and palmitoyl-CoA desaturase activity, processes that are at least partially regulated transcriptionally, mediate the altered lipid composition in ether lipid-deficient mutants. Phenotypic analysis demonstrated the importance of ether-linked lipids for optimal fertility, lifespan, survival at cold temperatures, and resistance to oxidative stress. These discoveries indicate that investigation of ether lipid synthesis and function in *C. elegans* will make important contributions to understanding the roles of ether lipids in human health and disease.

## MATERIALS AND METHODS

### Nematode strains

The wild-type *C. elegans* strain was N2, strains BX11 *fard-1(wa2)*, BX275 *fard-1(wa28)*, BX10 *ads-1(wa3)*, and BX259 *acl-7(wa20)* were isolated in a genetic screen carried out in the Browse laboratory (14) and outcrossed four times against wild-type before analysis. Unless otherwise stated, the *fard-1* strain used was BX275 *fard-1(wa28)*. The *ire-1(v33)* strain and the “million mutant” strains (VC) were obtained from the Center for Caenorhabditis Genetics Stock Center (St. Paul, MN). Nematodes were maintained on nematode growth media at 20°C seeded with *Escherichia coli* (OP50), unless otherwise stated (15). For RNAi experiments, nematode growth media was supplemented with 100 μg/ml ampicillin and 2 mM isopropyl β-D-1-thiogalactopyranoside, and seeded with the appropriate HT115 RNAi bacteria (16). The plasmid for *fard-1* feeding RNAi was obtained from the Ahringer RNAi library (16) and was sequenced before use. For *acl-7* RNAi and *ads-1* RNAi, feeding RNAi plasmids were constructed by cloning of *acl-7* and *ads-1* genomic sequences into the L4440 vector. Primers for *ads-1* RNAi cloning were (forward) GAAGATGTTCGCCGGATCCAGCTC and (reverse) GGTCAAGAGATTCGGAAGCTTCGTG; for *acl-7* cloning (forward) GAGTCAACTGGAGCTCTACTGAAAGC and (reverse) GAGCACAAGCTTGCTCAACACTTCTG. The PCR products and L4440 plasmid were digested with restriction enzymes *Bam*HI and *Hin*dIII (for *ads-1*) and *Sac*I and *Hin*dIII (for *acl-7*) before ligation and transformation into *E. coli* HT115.

### Whole genome sequencing

DNA was extracted from approximately 1,000 nematodes using a Qiagen DNeasy kit. Samples were sequenced using a MiSeq PE250 run with an expected coverage of ∼10× per sample. Reads were then cleaned with SeqyClean, mapped against the WBcel215.70 (http://jan2013.archive.ensembl.org/Caenorhabditis_elegans/Info/Index) using Bowtie 2 v2.1.0, and variants were called with the SAMtools package v0.1.19-44428cd. Variants were then filtered and annotated using an R program developed in-house.

### Lipid analysis

Fatty acid composition of young adult nematodes was determined by GC-MS, as previously described (14, 17). For lipid composition studies, lipids were extracted from three biological replicates, each containing approximately 10,000 young adult nematodes. Lipid extraction and separation of the triacylglycerol (TAG) and phospholipid fractions used a two solvent TLC protocol, as described previously (18). Stable isotope labeling of fatty acids was performed essentially as described (19). Briefly, equal amounts of bacteria grown in either Luria broth (^12^C media) or ISOGROW (98.5% ^13^C-enriched, Sigma) were mixed and plated onto agarose plates. For each sample, approximately 30,000 synchronized L1 nematodes prepared from hypochlorite treatment of gravid adults were added to the plates and grown for 48 h at 20°C. Lipids were extracted and fatty acids were analyzed as described (19).

### LC-MS/MS molecular species analysis

Ultra-performance LC molecular species separation and chip-based nanoelectrospray ionization (TriVersa Nanomate^®^; Advion, Ithaca, NY) were achieved as described in Tarazona, Feussner, and Feussner (20), including only the use of the ultra-performance LC gradient “B” combined with the negative ion mode. Phosphatidylethanolamine (PE) molecular species were detected with a 4000 QTRAP^®^ tandem mass spectrometer (AB Sciex, Framingham, MA) by monitoring: *i*) single reaction monitoring (SRM) transitions from [M-H]^−^ molecular ions to acyl chain-derived carboxylate fragments (supplementary Table 1); and *ii*) plasmalogen-specific SRM transitions as in (21), which targeted *sn-*1- and *sn*-2-related fragments in positive ion mode (supplementary Table 2). As an additional control, the identities of 17 ether lipids were confirmed by their accurate mass (supplementary Table 3). For this purpose, the post column flow was directed to an orthogonal time-of-flight mass spectrometer (LCT Premier; Waters Corp., Milford, MA) and analyzed as in (20). The identification criteria included: *i*) two complementary SRM transitions for diacyl-PE and 1-*O-*alk-1′-enyl-2-acyl-PE species, and one SRM transition for 1-*O-*alkyl-2-acyl-PE species; *ii*) retention time for all analyzed PE species; and *iii*) accurate mass analysis for 17 ether lipid molecular species. The chromatographic resolution of isobaric PE species was required for analyte identification. Due to the unavailability of primary standards and isotopically labeled internal standards for each of the targeted analytes, no absolute quantification was intended in this broad ranging LC-MS approach.

### Semi-quantitative and quantitative RT-PCR analysis

Nematodes were synchronized and harvested at L4 stage. RNA and cDNA were prepared as described (22). For semi-quantitative RT-PCR, the PCR cycles were as follows: *fat-6*, 22 cycles; *pod-2* and *cdc-42*, 25 cycles; *fat-5* and *fat-7*, 28 cycles; *elo-2*, 30 cycles. Real-time PCR assays were run and monitored with an ABI Prism 7000 sequence detection system (Applied Biosystems, Foster City, CA). The real-time PCR was conducted on at least three treatment groups with each individual treatment group at least in triplicate. Threshold values (Ct) for the gene of interest and a reference gene, *cdc-2*, were determined using ABI Prism SDS software version 1.1 (Applied Biosystems). The expression level of the gene of interest was evaluated using the 2^−(ΔΔCt)^ method (23). Supplementary Table 4 shows primer sequences used for the quantitative real-time RT-PCR.

### Physiological assays

#### Cold survival.

For each strain, approximately 100 synchronized L1 larvae were plated onto 12 6 mm NGM plates. Four plates of each strain were transferred to incubators set to 20°C, 15°C, and 10°C. The number of L4 and young adult worms were counted after 3 days of growth for the 20°C plates, 5 days of growth at 15°C, and 9 days of growth at 10°C. To ensure that the 10°C worms were large enough to visualize, after 9 days of growth at 10°C, the plates were transferred to 20°C before counting viable worms. The number of viable worms at 15°C and 10°C were normalized to the number at 20°C to calculate survival.

#### Brood size.

For analysis of total progeny produced per worm, eight to ten L4 larvae were transferred individually to fresh NGM plates seeded with *E. coli* strain OP50. Worms were transferred daily until they did not produce any more progeny. Two days after removal of the adult, the live progeny of each individual were counted.

#### Lifespan and stress analysis.

Lifespan assays were conducted at 20°C on solid NGM media seeded with *E. coli* (OP50) using standard protocols and were replicated in at least three independent experiments. For lifespan assays, worms were moved to new assay plates every 1–2 days to prevent contamination of plates with their offspring. To assess the survival of the worms, the animals were prodded with a platinum wire every 1–2 days, and those that failed to respond were scored as dead. All lifespan data are available in supplementary Table 5, including sample sizes. Animals were assigned randomly to the experimental groups. Worms that ruptured, bagged (that is, exhibited internal progeny hatching), or crawled off the plates were censored. *P* values were calculated using the log-rank (Cox-Mantel) test. Lifespan data were analyzed using GraphPad Prism 6 and OASIS (online application for the survival analysis of lifespan assays performed in aging research) (24).

To assess sensitivity to acute oxidative stress, at least 100 young adult worms that were grown on NGM plates were transferred to freshly prepared plates containing 200 mM paraquat or 14.6 mM tert-butyl peroxide (tBOOH) (25, 26). Worms were scored every 2 h as alive or dead. For endoplasmic reticulum (ER) stress assays, at least 100 L4 worms that were grown on NGM plates were transferred to 30 mg/ml tunicamycin plates and were scored as alive or dead every 24 h (27). For stress assays, growth of worms and assays were performed at 20°C. Survival on paraquat, tBOOH, and tunicamycin was plotted in GraphPad Prism and curves were compared using a log-rank (Cox-Mantel) test.

## RESULTS

### Isolation of *C. elegans* ether lipid-deficient mutants

**Fig. 1.**
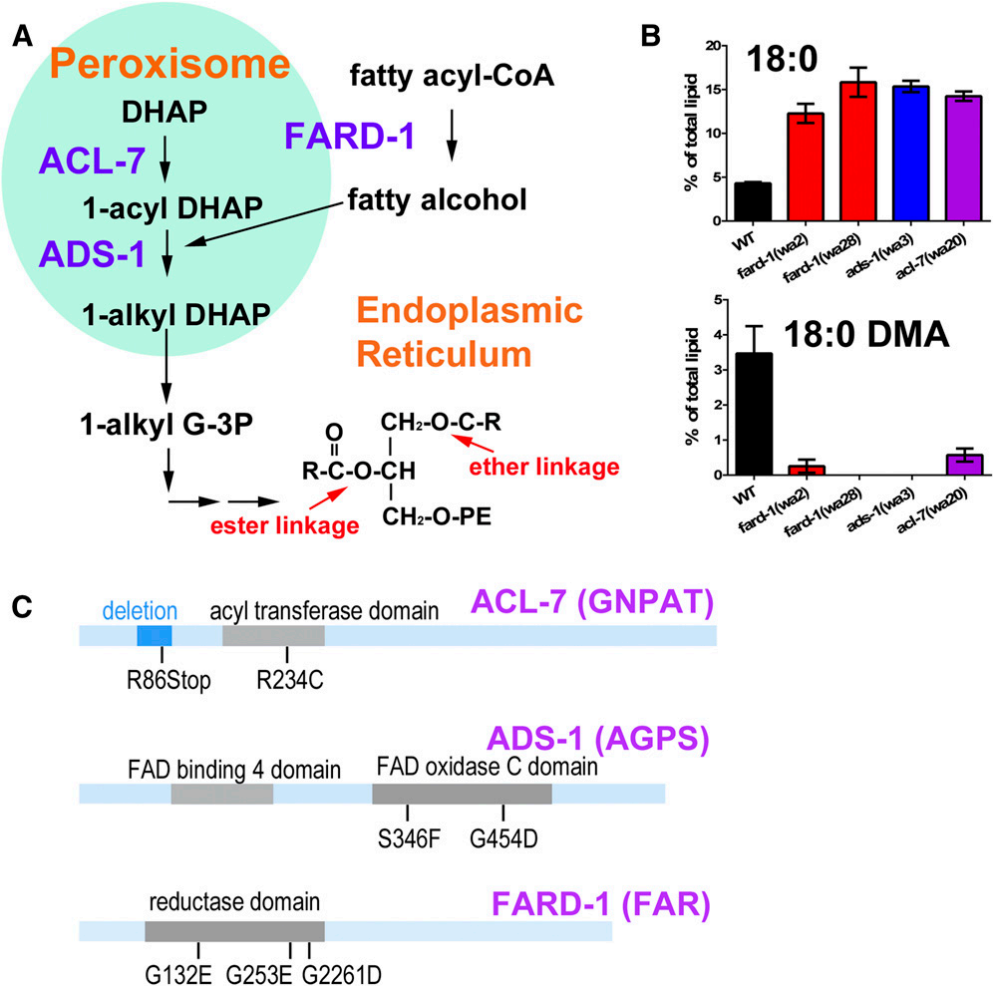
Identification of ether lipid-deficient *C. elegans* mutants. A: Ether lipid synthesis begins in the peroxisome with three key biochemical reactions. The acyltransferase, ACL-7 (GNPAT in mammals), adds an acyl group to dihydroxyacetonephosphate (DHAP). The fatty acyl-CoA reductase, FARD-1 (FAR1 in mammals), associated with peroxisomal membrane, supplies the fatty alcohols, which are used by ADS-1 (AGPS in mammals) to replace the acyl group, forming 1-aklyl DHAP, which leaves the peroxisome and serves as the basis for the formation of ether-linked phospholipids in the ER. B: GC-MS analysis reveals that *C. elegans* strains carrying mutations in *fard-1*, *ads-1*, and *acl-7* accumulate high levels of stearic acid (18:0) and reduced or undetectable levels of 18:0 DMA, an indirect measure of ether-linked lipids. For both fatty acids, all mutant strains showed significant difference from wild-type using one-way ANOVA and Dunnett’s multiple comparisons test. C: Schematic representation of ACL-7, ADS-1, and FARD-1 showing the molecular nature of mutations that lead to depletion of 18:0 DMA and increased levels of stearic acid (18:0) in *C. elegans*.

To study the relationships between specific lipid species and biological functions, we performed a genetic screen for *C. elegans* mutants with altered fatty acid composition. We successfully identified mutants in the fatty acid desaturation and elongation pathway required for the biosynthesis of PUFAs (14). Several mutant lines isolated in this screen (*wa2*, *wa3*, *wa20*, *wa28*) contained 3- to 4-fold higher saturated fat than wild-type, mostly in the form of high levels of stearic acid (18:0). After we ruled out that these strains did not carry mutations in the delta-9 desaturase genes (*fat-5*, *fat-6*, or *fat-7*) (17, 22), or in known transcriptional regulators of these genes (*sbp-1*, *nhr-80*, *nhr-49*, *mdt-15*) (17, 28–30), we mapped two of the mutants to determine their chromosomal location and used whole genome sequencing to sequence all four of the mutants. We found that the four mutations were in three genes that encode proteins necessary for the first steps of ether lipid synthesis. These genes are *fard-1*, FAR, *acl-7*, the peroxisomal DHA acyltransferase (GNPAT), and *ads-1*, the peroxisomal alkyl-DHAP synthase (AGPS) (**Fig. 1A**). Loss-of-function mutations in the human homologs of *ads-1* (AGPS) and *acl-7* (GNPAT) are associated with the diseases RCDP types 2 and 3 (7).

After further analysis of the fatty acid composition of these mutants, we noticed that the putative ether lipid biosynthetic mutants not only contained relatively high amounts of stearic acid (18:0), but also lacked a fatty acid derivative that we identified by MS as 18:0 dimethylacetal (DMA) (Fig. 1B, supplementary Table 6). This fatty acid derivative corresponds to the 18:0 fatty acyl component of PE-plasmalogen, the ether lipid containing a delta-1 *cis* double bond. This mass spectrum of this fatty acid derivative is distinct among the fatty acid methyl esters that are derived from conventional diacyl phospholipids (31). Even though the alkyl fatty acids were not cleaved from the ether lipids during our GC-MS derivatization protocol, the alkenyl ether lipids were cleaved and converted to 18:0 DMA, indicating the presence of alkenyl or plasmalogen ether lipids. To confirm the identities of our mutant lines, we fed wild-type *C. elegans* RNAi constructs corresponding to the *fard-1*, *acl-7*, and *ads-1* genes. This resulted in the accumulation of 18:0 and depletion of 18:0 DMA, confirming the identification of these genes as being causative for the fatty acid phenotype (supplementary Fig. 1, supplementary Table 6). In addition, we analyzed the fatty acid composition of putative ether lipid biosynthesis mutants from the *C. elegans* million mutant project (32). Several of these lines, including two putative null alleles (deletion or premature stop codons) showed high saturated fat and lack of 18:0 DMA, indicating lack of ether lipid biosynthetic capability. The specific mutations are summarized in Fig. 1C and **Table 1**. All of the strains that showed the high 18:0 and low 18:0 DMA phenotypes contained point mutations in conserved functional domains, although not all mutations in the conserved domains manifested the altered fatty acid phenotype. We did not identify any mutants that showed only low 18:0 DMA without high 18:0. Therefore, in this set of mutants, increases in 18:0 are always associated with mutant strains that are unable to synthesize ether-linked lipids.

**TABLE 1.** *C. elegans* strains containing mutations in ether lipid biosynthesis genes

Gene	Strain Name	Allele	AA change	Conserved Domain	GC/MS Phenotype	Mutagen
acl-7	VC20564	gk158076	G315R	—	Wild-type	EMS
acl-7	VC30016	gk158078	P287S	—	Wild-type	ENU
acl-7	VC30097	gk158077	E301K	—	Wild-type	ENU
acl-7	VC40074	gk158071, gk158074	T642I, D338N	—	Wild-type	ENU + EMS
acl-7	VC20381	gk158075	N365K	—	Wild-type	EMS
**acl-7**	**BX259**	**wa20**	**R234C**	**Acyl transfer domain**	**High 18:0, slight 18:0 DMA**	**EMS**
**acl-7**	**VC40979**	**gk910077**	**R86 Opal**	**Acyl transfer domain**	**High 18:0, no 18:0 DMA**	**ENU + EMS**
acl-7	VC20532	gk341012	P241S	Acyl transfer domain	Wild-type	EMS
**acl-7**	**VC40643**	**gk961438**	**Deletion AA68-92**	—	**High 18:0, no 18:0 DMA**	**ENU + EMS**
ads-1	VC20023	gk341012	R182Q	FAD binding 4	Wild-type	EMS
**ads-1**	**VC20752**	**gk391946**	**S246F**	**FAD oxidase C**	**High 18:0, no 18:0 DMA**	**EMS**
ads-1	VC20757	gk393652	G539D	FAD oxidase C	Wild-type	EMS
ads-1	VC40662	gk751286	P489S	FAD oxidase C	Wild-type	ENU + EMS
**ads-1**	**BX10**	**wa3**	**G454D**	**FAD oxidase C**	**High 18:0, no 18:0 DMA**	**EMS**
**fard-1**	**VC20061**	**gk164361**	**G132E**	**Reductase/NAD binding**	**High 18:0, no 18:0 DMA**	**EMS**
**fard-1**	**BX11**	**wa2**	**G253E**	**Reductase/NAD binding**	**High 18:0, slight 18:0 DMA**	**EMS**
**fard-1**	**BX275**	**wa28**	**G261D**	**Reductase/NAD binding**	**High 18:0, no 18:0 DMA**	**EMS**
fard-1	VC20611	gk354624	A235T	Reductase/NAD binding	Wild-type	EMS
fard-1	VC30262	gk450456	D98G	Reductase/NAD binding	Wild-type	ENU
fard-1	VC40103	gk463717	R513W	—	Wild-type	EMS + ENU
Bold rows indicate mutations that affect ether lipid synthesis. EMS, ethyl methanesulfanate; ENU, *N*-ethyl-*N*-nitrosourea.						

### Characterization of PE species with alkyl and alkenyl ether lipids

**Fig. 2.**
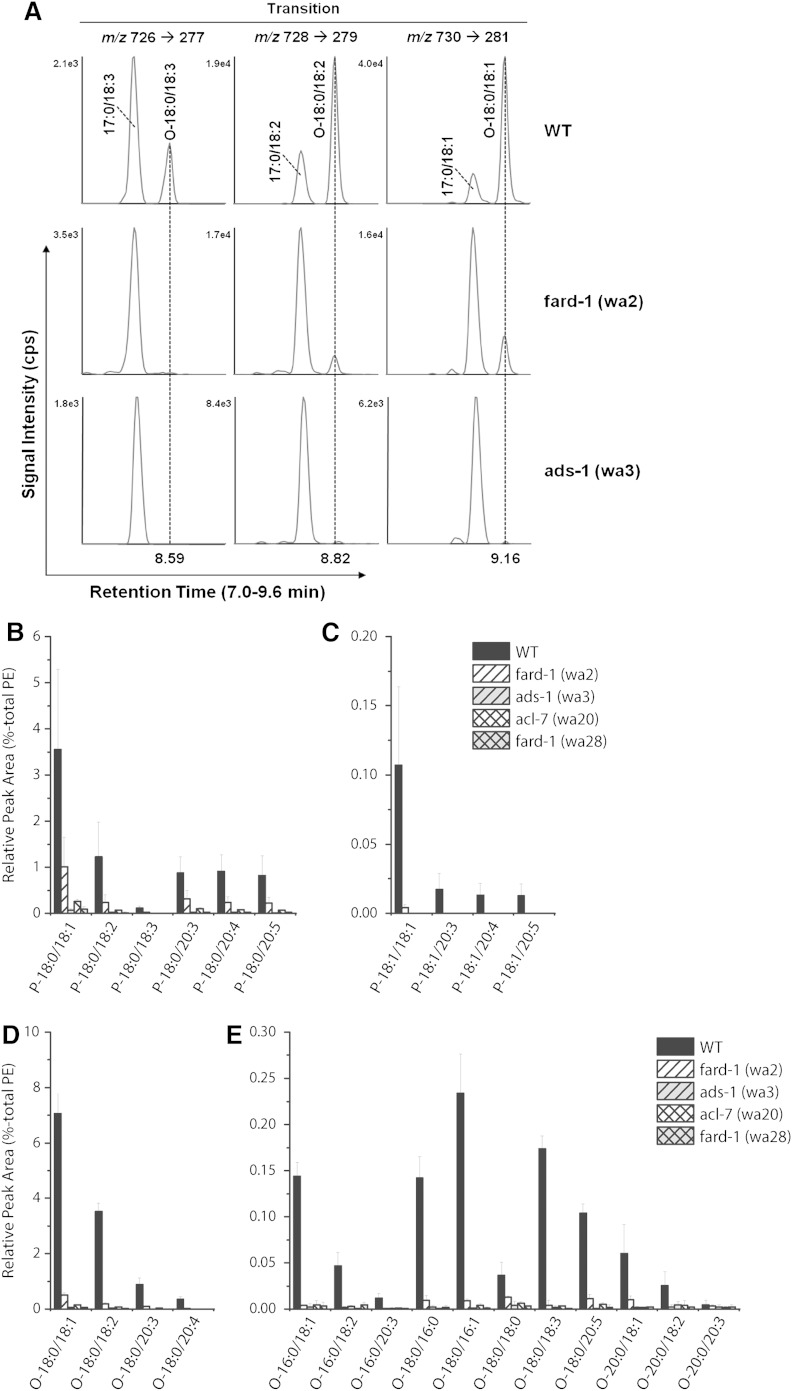
LC-MS/MS analysis of ether-linked lipids. A: Chromatographic separation of isobaric PE molecular species. Each of the three SRM transitions from [M-H]^−^ PE molecular ions to fatty acyl-derived carboxylate fragments gives rise to two baseline-separated chromatographic peaks. The first peak corresponds to a diacyl-PE species containing 17:0, whereas the second peak corresponds to a 1-*O-*alkyl-2-acyl-PE species containing O-18:0. All three 1-*O-*alkyl-2-acyl-PE species are depleted in the *fard-1* and *ads-1* mutant strains. Representation of relative amounts of major (B) and minor (C) alkyl (O) species of PE lipids and major (D) and minor (E) alkenyl (P) species of PE lipids.

While the loss of 18:0 DMA is predictive for ether lipid biosynthetic defects, this fatty acid only represents one of the alkenyl fatty acids that are the plasmalogen components of ether lipids. Therefore, we used a more sensitive LC-MS/MS technique to characterize ether-linked lipids in *C. elegans* wild-type and mutants. We identified the diacyl, alkyl, and alkenyl derivatives of phosphatidylcholine (PC) and PE. Because the PC fraction contained only trace amounts of alkyl or alkenyl lipids, we focused our analysis on the PE lipids. The chromatographic resolution of isobaric PE species was required for analyte identification (**Fig. 2A**). Due to the unavailability of primary standards and isotopically labeled internal standards for each of the targeted analytes, we compared the relative LC-MS lipid profiles, which revealed striking differences between wild-type and mutant strains (Fig. 2).

In wild-type extracts, we identified 15 PE species containing alkyl 18:0 or 16:0 fatty acids (Fig. 2B, C; supplementary Table 7), and 10 PE species containing alkenyl 18:0 or 18:1 (Fig. 2D, E; supplementary Table 7). All of these species were absent or severely depleted in the *fard-1*, *ads-1*, and *acl-7* mutant strains. In contrast, the diacyl PE species consisted of 103 distinct lipids (supplementary Figs. 2, 3). We found that all 14 of the diacyl PE species containing stearic acid (18:0) were increased in all of the ether lipid-deficient mutants relative to wild-type controls and seven of the PE species containing arachidic acid (20:0) were increased in the mutants relative to wild-type controls (supplementary Figs. 2, 3; supplementary Table 7). Thus, saturated diacyl PE species accumulate in the ether lipid-deficient mutant strains.

### Ether lipid-deficient strains *fard-1*, *ads-1*, and *acl-7* have higher levels of saturated fat in all lipid classes than wild-type

**Fig. 3.**
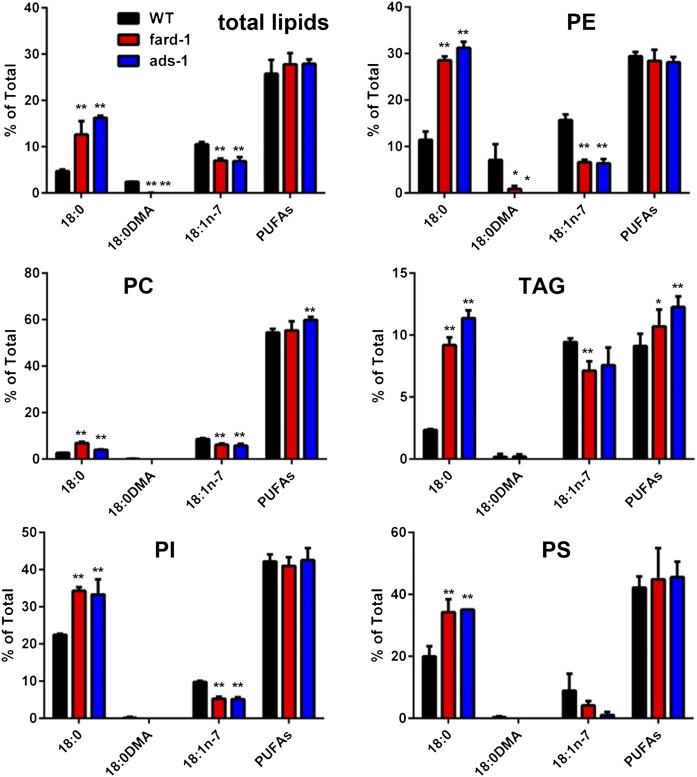
Accumulation of 18:0 and reduction of 18:1(n-7) in all lipid classes in ether lipid-deficient mutants, *ads-1(wa3)* and *fard-1(wa2)*. Shown is the average fatty acid composition relative to total fatty acids. Error bars are standard deviation, *P < 0.05, **P < 0.01.

In two of the mutant strains, *ads-1(wa3)* and *fard-1(wa2)*, we conducted further lipid separation and fatty acid composition analysis. We examined the fatty acid composition in total lipids, TAGs, PC, PE, phosphatidylserine (PS), and phosphatidylinositol (PI). We found that levels of TAGs did not significantly vary from wild-type, indicating that lack of ether-linked lipids does not affect fat accumulation (**Table 2**). However, the ether lipid-deficient mutants did show altered ratios of PC and PE, with the mutants accumulating relatively more fatty acids in PE than PC (Table 2). After examining the fatty acid composition of each lipid fraction, we found that levels of PUFAs were normal in all lipid classes in the mutant strains, yet all lipid classes showed greatly increased stearic acid (18:0) and reduced *cis*-vaccenic acid (18:1n-7), even though none of the lipid classes, except for PE, accumulated appreciable amounts of ether-linked lipids (**Fig. 3**, supplementary Table 8). Therefore, fatty acid composition changes that apparently compensate for lack of ether lipids occur in all classes of *C. elegans* lipids, not just in PE.

**TABLE 2.** Relative percentage of phospholipid classes and TAGs in wild-type (N2), *fard-1(wa2)*, and *ads-1(wa3)* mutants

	Relative Percent of Phospholipids					
	PC/PL (%)	PE/PL (%)	PI/PL (%)	PS/PL (%)	PC:PE	TAG/TL (%)
Wild-type	46.2 (1.7)^a^	40.8 (1.5)^a^	6.5 (0.4)^a^	3.9 (0.7)^a^	1.13^a^	50.0 (0.3)^a^
*fard-1(wa2)*	39.6 (1.1)^b^	46.7 (1.5)^b^	6.7 (0.4)^a^	4.8 (0.8)^a^	0.85^b^	48.6 (1.2)^a^
*ads-1(wa3)*	36.4 (0.3)^b^	46.2 (0.7)^b^	7.5 (0.2)^b^	5.2 (0.4)^a^	0.79^b^	48.4 (0.7)^a^

Values are the average (SD) from three independent lipid extractions of young adult stage *C. elegans*. Those not sharing a common letter within the same column differ (*P* < 0.05). PL, phospholipids; TL, total lipids.

### De novo fatty acid synthesis is increased in ether lipid-deficient mutants

**Fig. 4.**
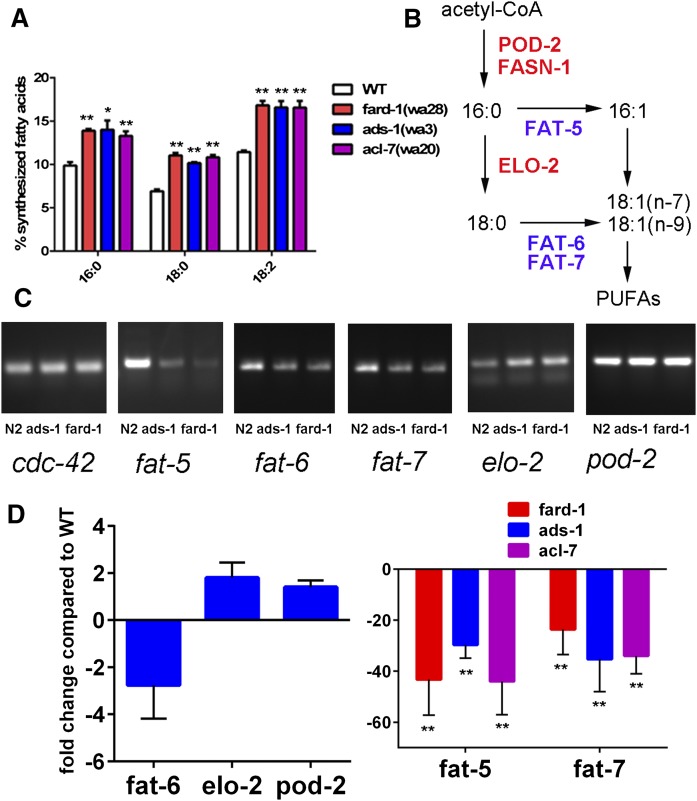
*C. elegans* mutants lacking ether lipids compensate by increasing de novo fatty acid synthesis and reducing fatty acid desaturation. A: Stable isotope labeling experiments demonstrate increased de novo synthesis of fatty acids in ether lipid-deficient mutants. The difference in the mean percentage of synthesized fatty acids is significant for all three mutants compared with wild-type for all three fatty acids measure. Error bars depict SEM, *P < 0.05, **P < 0.01. B: Abbreviated pathway of fatty acid synthesis, elongation, and desaturation. C: Semi-quantitative RT-PCR analysis showing reduced expression of *fat-5*, *fat-6*, and *fat-7* and increased expression of *elo-2* in the ether lipid-deficient mutants. Expression of the control gene, *cdc-42*, does not change. D: Confirmation of by quantitative real-time RT-PCR shows decreased expression of delta-9 desaturases, *fat-5* and *fat-7*, and a trend toward decreased expression of *fat-6* and increased expression of *elo-1* and *pod-2* in the ether lipid-deficient mutant strains compared with wild-type. Error bars depict SEM, **P < 0.01.

To investigate the mechanism of the lipid composition changes that occur in the absence of ether lipids, we first examined whether ether lipid mutants underwent changes in fatty acid synthesis by performing a stable isotope labeling experiment combined with GC-MS analysis to measure de novo fatty acid synthesis (19). By feeding worms a mixture of ^13^C-labeled and unlabeled bacteria, we could determine from the mass spectra whether particular fatty acids were obtained from the diet (uniformly labeled or unlabeled carbons) or were synthesized de novo (fatty acyl species containing a mixture of labeled and unlabeled carbons). We found a 33–50% increase in de novo synthesized fatty acids in *fard-1*, *ads-1*, and *acl-7* mutant strains compared with wild-type (**Fig. 4A**). Thus, a higher percentage of fatty acids, including 16:0, 18:0, and 18:2, are synthesized de novo from acetyl-CoA, rather than being absorbed and modified from dietary fatty acids obtained from digested *E. coli* membranes. This indicates that increased de novo fatty acid synthesis occurs in response to ether lipid deficiency, although, interestingly, the increased de novo synthesis does not result in increased fat (TAG) accumulation (Table 2).

### Delta-9 desaturase genes are distinctly downregulated in ether lipid-deficient *C. elegans*

To investigate whether the increased de novo fat synthesis and increased saturated fat content might be due to transcriptional changes in the ether lipid-deficient mutants, we used reverse transcriptase-PCR to examine gene expression changes of key genes involved in fatty acid synthesis, elongation, and desaturation (Fig. 4B). Semi-quantitative RT-PCR experiments indicate that the *fard-1* and *ads-1* mutants have reduced expression of the three delta-9 desaturase homologs, *fat-5*, *fat-6*, and *fat-7*, which encode fatty acid desaturase enzymes that catalyze the formation of a double bond in a saturated fatty acid (22) (Fig. 4C). The *fat-5* gene encodes a palmitoyl-CoA desaturase, while *fat-6* and *fat-7* encode stearoyl-CoA desaturases (33). Reduced expression of these delta-9 desaturases is consistent with increased saturated fat content of these mutants. In addition, the mutant worms appear to exhibit an increase in *elo-2*, the gene encoding the 16:0 fatty acid elongase (34), and a slight increase in expression of *pod-2*, the gene encoding acyl-CoA carboxylase (35), the rate limiting enzyme for de novo fatty acid synthesis (Fig. 4C). Real-time quantitative RT-PCR using RNA isolated from wild-type and the ether lipid-deficient mutants showed a highly significant decrease in expression for *fat-5* and *fat-7* in *fard-1*, *ads-1*, and *acl-7* mutants compared with wild-type, while gene expression changes in *ads-1* mutants compared with wild-type for the genes *fat-6*, *elo-2*, and *pod-2* showed a similar trend, as observed with the semi-quantitative RT-PCR results, but it was not significant (Fig. 4D). Together these data indicate that *C. elegans* responds to the lack of ether lipids by upregulating fatty acid synthesis pathways and downregulating fatty acid desaturase pathways. Our data do not support a strong role for transcriptional upregulation of *pod-2* to support increased de novo fatty acid synthesis, but show that repression of transcription of the fatty acid desaturases likely mediates their reduction of activity. The changes in fatty acid synthesis, elongation, and desaturation ultimately lead to increased 18:0 accumulation.

### Ether lipid-deficient nematodes exhibit cold sensitivity, reduced fecundity, short lifespan, and sensitivity to oxidative stress

**Fig. 5.**
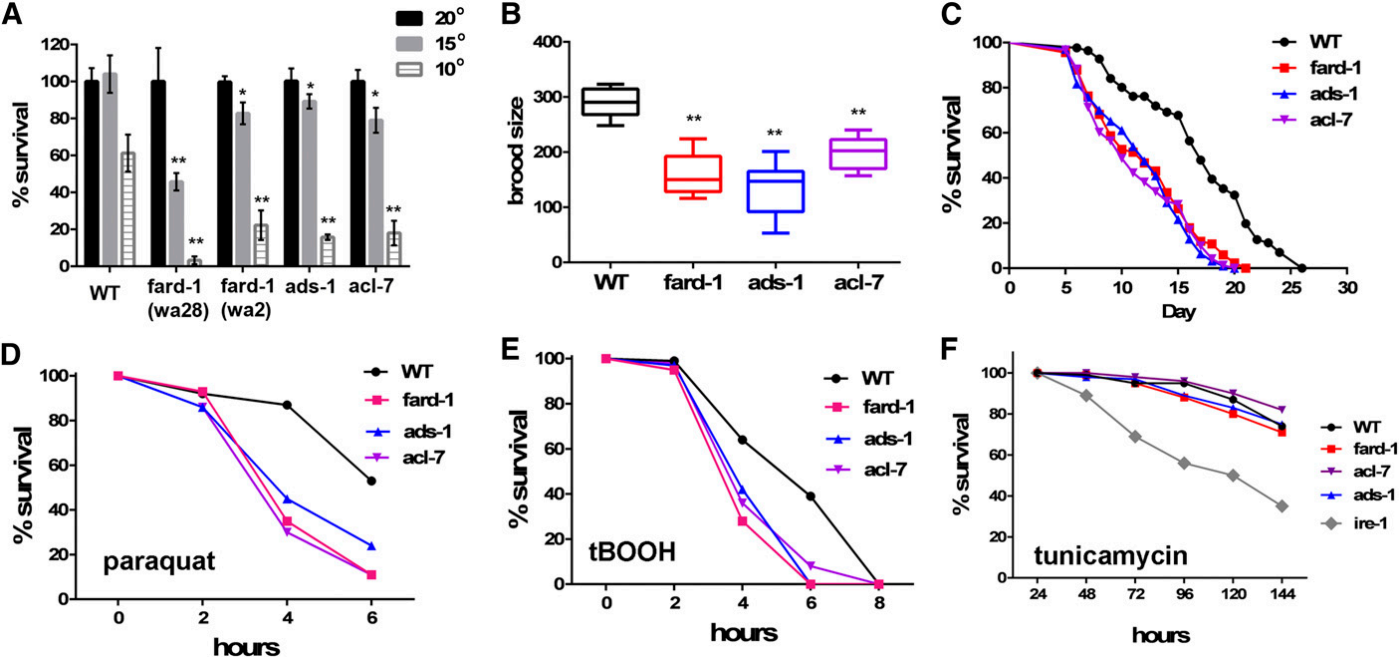
Physiological consequences of ether lipid deficiency. A: Ether lipid-deficient mutants show reduced survival when grown at 15°C, and fail to survive at 10°C. Two-tailed *t*-tests were used to determine the significance between the mean cold survival of each mutant strain compared with wild-type at the same temperature. *P < 0.05, **P < 0.001. B: Ether lipid-deficient mutants have a reduced number of viable progeny at 20°C. Graphs show the range and average number of progeny in eight to ten individuals of each genotype. The brood size of each mutant is significantly different than wild-type, ** *P* < 0.0001 as determined by one-way ANOVA and Dunnett’s multiple comparison test. C: Lifespan of ether lipid-deficient mutants at 20°C is shorter than wild-type, *P* < 0.0001 as determined by log-rank (Cox-Mantel) test. D, E: Ether lipid-deficient mutants show reduced survival in the presence of acute oxidative stress. For both oxidants, *P* < 0.001 as determined by log-rank (Cox-Mantel) test. F: Ether lipid-deficient mutants are not more sensitive to ER stress than wild-type.

Humans born with mutations in the genes encoding AGPS, GNPAT, and FAR1 die as young children, with severe growth and neurological defects (6, 8). In contrast, *C. elegans ads-1*, *acl-7*, and *fard-1* mutants are viable and grow only slightly more slowly than wild-type at 20°C. However, the *C. elegans* ether lipid mutants show reduced survival at the low growth temperature of 15°C, and do not survive at 10°C, which is the lowest temperature in which wild-type *C. elegans* is capable of survival (**Fig. 5A**). Furthermore, all of the ether lipid mutants produced fewer progeny than wild-type at the normal growth temperature of 20°C, with the average brood size of 135–200 progeny per worm in the mutant strains, compared with the wild-type brood size of 290 (Fig. 5B). At 20°C, both the mean lifespan and the maximum lifespan of all three ether lipid-deficient strains were significantly shorter than wild-type. The median lifespan of the ether lipid-deficient mutant strains ranged from 21 to 43% shorter than WT (Fig. 5C, supplementary Table 5).

A major role of ether lipids, particularly the PE plasmalogens, frequently discussed in the literature is their ability to protect cells against reactive oxygen species, reviewed in (5). We tested whether the *C. elegans* ether lipid-deficient mutants were more sensitive to oxidative stress by placing young adult nematodes onto freshly made plates containing relatively high concentrations of methyl viologen dichloride (also known as paraquat) and tBOOH. These chemicals generate reactive oxygen species, which damage cellular proteins, nucleic acids, and lipids. At the concentrations used in this study, tBOOH causes 100% death in wild-type after 8 h of exposure. The ether lipid-deficient mutants, however, die even more rapidly, with nearly 100% death by 6 h of exposure. Similarly, paraquat causes 50% death in wild-type after 6 h of exposure, while at the same time point, 80–90% of the mutants have died (Fig. 5D, E). In contrast, the ether lipid-deficient mutants did not show increased sensitivity to tunicamycin, which elicits ER stress (Fig. 5F), indicating that mechanisms to deal with ER stress are intact, in spite of a lack of ether lipids. Overall, ether lipid deficiency affects the physiology of *C. elegans*, with reduced reproduction, inability to survive low growth temperatures, reduced lifespan, and reduced tolerance for oxidative stress.

## DISCUSSION

Despite their widespread abundance in nature and their importance to human health, ether lipids are relatively understudied because of the technical challenges of studying their synthesis and function in mammals, and their specific cellular roles are not understood. We isolated *C. elegans* strains carrying mutations in genes encoding proteins that catalyze the first three biosynthetic steps of the ether lipid biosynthetic pathway. Mutations in genes encoding the first three steps of ether lipid biosynthesis all had very similar phenotypes and lipid profiles. Two of the genes encode peroxisomal proteins (ACL-7/GNPAT and ADS-1/AGPS) required for the first two steps of ether lipid synthesis genes. The third gene corresponds to fatty acyl-CoA reductase (FARD-1/FAR1), which supplies the fatty alcohol for the synthesis reaction. FAR1 was recently shown to be an integral peroxisomal membrane protein, the stability of which is regulated by plasmalogen levels (36).

Analysis of the ether lipid-deficient mutant strains demonstrates that the inability to synthesize ether lipids induces remarkable remodeling in all *C. elegans* lipids, leading to greatly increased saturated fatty acids, mostly in the form of stearic acid (18:0), in all major lipid classes, including TAGs. Previously, we documented the ability for *C. elegans* to undergo compensation when single delta-9 desaturases were mutated (17). We found substantial upregulation of *fat-5* and *fat-7* genes when the highest expressed homolog, *fat-6*, was mutated. In the present study, we found that the *fat-5* and *fat-7* genes were essentially turned off in strains carrying mutations in the ether lipid biosynthetic genes. Lack of delta-9 desaturation leads to the accumulation of its substrate, stearic acid (18:0). Previous studies indicate that membranes in skin cells cultured from patients deficient in ether lipid synthesis were more fluid than membranes from control skin cells (37). Biophysical studies showed that the lack of the carbonyl oxygen at position *sn*-1 in ether lipids allows stronger intermolecular hydrogen-bonding between headgroups of lipids (38). Thus, increasing the level of saturated fatty acids in membranes likely compensates for the deficiency of ether lipids, by ensuring more membrane rigidity due to the saturated fatty acyl groups.

The stable isotope labeling experiments demonstrated that the ether lipid-deficient mutants exhibit increased de novo fatty acid synthesis (Fig. 4A). However, we only showed minor changes in transcript levels for *elo-2* and *pod-2* enzymes. In mammals, fatty acid synthesis is regulated posttranscriptionally by allosteric regulation or by phosphorylation of acetyl-CoA carboxylase (39). It is likely that these types of regulation contribute to the increased de novo fatty acid synthesis in the ether lipid-deficient mutants. Interestingly, a recent study showed that human cells supplemented with the ether lipid precursor, hexadecylglycerol, showed major changes in cellular phospholipid species (40). Together with our work, these studies demonstrate that altering the composition of ether lipids in cells results in widespread compensatory regulation of fatty acid composition and other lipid species.

We found that ether lipid-deficient *C. elegans* strains were sensitive to oxidative stress incurred by at least two chemicals, methyl viologen and tBOOH (Fig. 5D, E). Plasmalogen ether lipids are thought to act as cellular antioxidants, because oxidation of the vinyl ether bond is thought to prevent the oxidation of nearby PUFAs, and products of plasmalogen degradation do not propagate lipid oxidation (5, 41). Our results are consistent with this hypothesis. Interestingly, even though disruption of lipid homeostasis incurs an ER stress response (27), the ether lipid-deficient strains do not appear to be hypersensitive to tunicamycin treatment, which elicits ER stress.

Depletion of ether lipids in mice causes male infertility, defects in eye development, cataracts, and optic nerve hypoplasia (42). Human patients born with mutations in genes encoding AGPS, GNPAT, and FAR1 show severe growth and neurological abnormalities, and do not survive past childhood (6, 8). Unlike these mammals, ether lipid-deficient nematodes are viable and fertile, making them an attractive model for future studies of ether lipid function. However, the ether lipid-deficient nematodes are not as fit as wild-type, and they accumulate high levels of saturated fatty acids that may have detrimental effects. Similarly, mammalian models of ether lipid deficiency also show compensatory changes in phospholipid species, including increases in PE and PC species containing saturated fatty acids (10, 43). Future studies will be needed to determine whether the reduced fecundity, short lifespan, cold sensitivity, and oxidative stress sensitivity are due to the lack of ether lipids or, possibly, secondary effects of the high saturated fat content of nematode lipids. Overall, ether lipid-deficient nematodes promise to be useful models for determining specific biological roles for ether lipids, which can be studied using *C. elegans* on a cellular, tissue, or a whole organism level.

## Supplementary Material

Supplemental Data
