# Supplementary material for: A Caenorhabditis elegans model for ether lipid biosynthesis and function
Source: J Lipid Res. 2016 Feb;57(2):265–75. doi: 10.1194/jlr.M064808 (PMC4727422; doi:10.1194/jlr.M064808)
Supplement: Supplemental Data [file supp_57_2_265__index.html]

A Caenorhabditis elegans model for ether lipid biosynthesis and function — Supplemental Data 

# A *Caenorhabditis elegans* model for ether lipid biosynthesis and function

## Supplemental Data

- Supplemental Figures S1, S2, S3 (.pdf, 347 KB) - Figure S1. GC-MS analysis of worms treated with feeding RNAi corresponding to fard-1, ads-1, and acl-7. Figure S2. LC-MS/MS analysis of even-chained diacyl PE species in young adult C. elegans. Figure S3. LC-MS/MS analysis of odd-chained diacyl PE species in young adult C. elegans.
- Supplemental Table 1 (.pdf, 265 KB) - Single reaction monitoring (SRM) transitions of PE species.
- Supplemental Table 2 (.pdf, 162 KB) - Single reaction monitoring (SRM) transitions of plasmalogen-specific fragments.
- Supplemental Table 3 (.pdf, 266 KB) - Identity confirmation of 17 phosphatidylethanolamine ether lipids by untargeted ultra performance liquid chromatography time-of-flight (UPLC-TOF) analysis.
- Supplemental Table 4 (.pdf, 184 KB) - Primers used for quantitative real-time RT-PCR
- Supplemental Table 5 (.pdf, 187 KB) - Lifespan analysis summary.
- Supplemental Table 6 (.pdf, 235 KB) - Fatty acid composition of ether lipid-deficient mutant strains and RNAi treated strains as determined by GC-MS.
- Supplmental Table 7 (.xlsx, 20 KB) - PE profiling in C. elegans strains
- Supplemental Table 8 (.xlsx, 21 KB) - Fatty Acid Composition of isolated Lipids Classes
