# Supplementary material for: A Caenorhabditis elegans model for ether lipid biosynthesis and function
Source: J Lipid Res. 2016 Feb;57(2):265–75. doi: 10.1194/jlr.M064808 (PMC4727422; doi:10.1194/jlr.M064808)

Shi et. al. Supplemental Figures

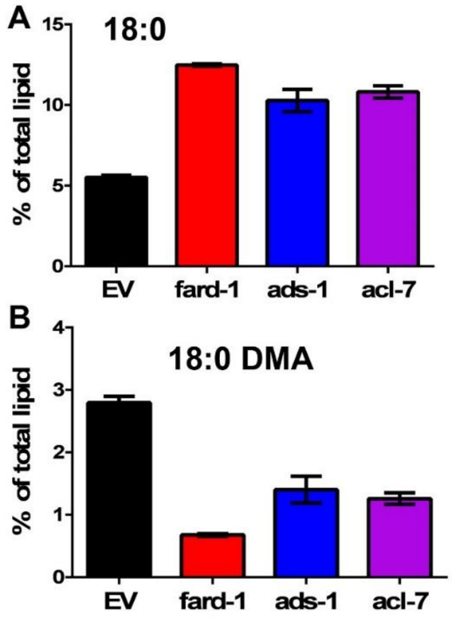

Figure S1. GC-MS analysis of worms treated with feeding RNAi corresponding to *fard-1*, *ads-1*, and *acl-7* show increased 18:0 (A) and decreased 18:0 DMA (B), similar to mutant strains. For both fatty acids, all mutant strains showed significant difference from the empty vector control using 1-way ANOVA and Dunnett's multiple comparisons test.

Figure S2. LC-MS/MS analysis of even-chained diacyl PE species in young adult *C. elegans*. Left graph shows the fold change in specific lipid species compared to wild type. Right graph shows the relative peak area of each species as a percentage of PE.

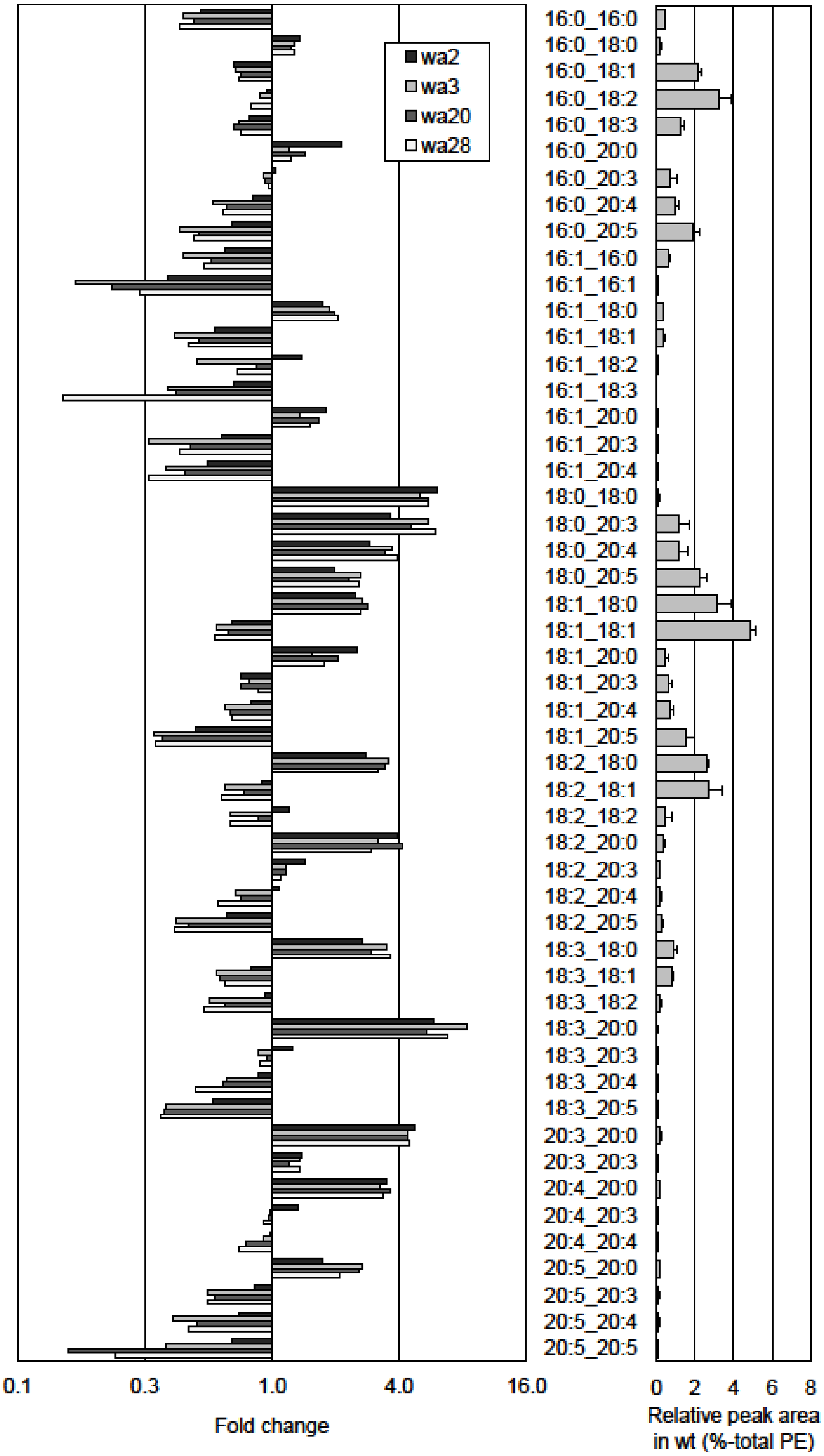

Figure S3. LC-MS/MS analysis of odd-chained diacyl PE species in young adult *C. elegans*. Left graph shows the fold change in specific lipid species compared to wild type. Right graph shows the relative peak area of each species as a percentage of PE.

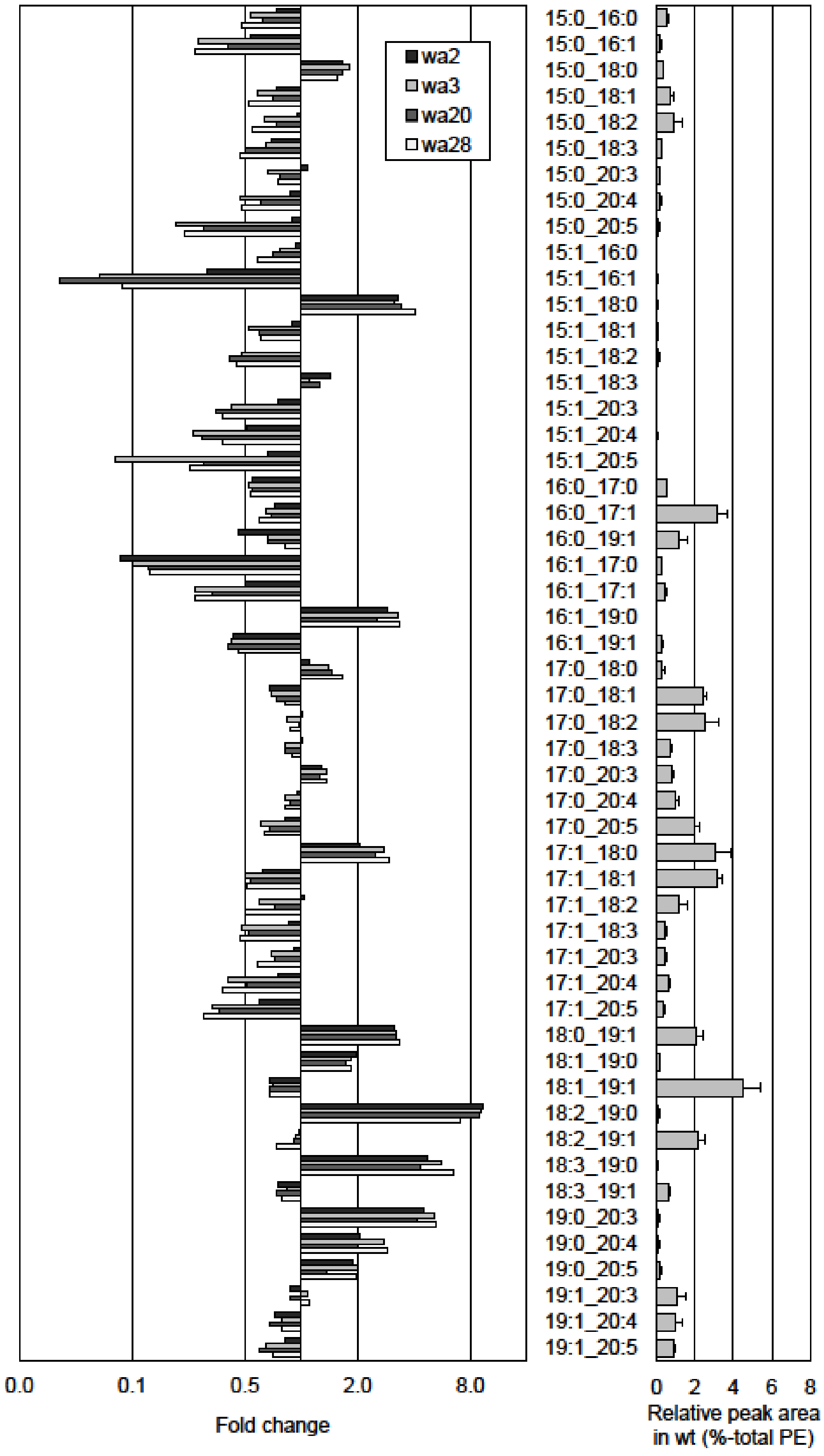

Supplement: Supplemental Data [file 10.1194_M064808_jlr.M064808-1.pdf]
