# Supplementary material for: A Caenorhabditis elegans model for ether lipid biosynthesis and function
Source: J Lipid Res. 2016 Feb;57(2):265–75. doi: 10.1194/jlr.M064808 (PMC4727422; doi:10.1194/jlr.M064808)
Supplement: Supplemental Data [file 10.1194_M064808_jlr.M064808-2.pdf]

Table S1. Single reaction monitoring (SRM) transitions from [M-H]$^-$ molecular ions to acyl chain derived carboxylate fragments. Transition name includes the lipid class phosphatidylethanolamine (PE), followed by the targeted apparent species in brackets. The acyl chain targeted in each transition is noted after an underscore. "O-" indicates apparent 1-O-Alkyl-2-acyl-PE species. "P-" indicates apparent 1-O-Alk-1'-enyl-2-acyl-PE species. The vinyl ether double bond of 1-O-Alk-1'-enyl-2-acyl-PE species was taken into account for the apparent species notation.

Precursor ion *m*/*z*	Fragment ion *m*/*z*	Transition name
672.5	239.2	PE(31:2)_15:1
672.5	253.2	PE(31:2)_16:1
674.5	239.2	PE(31:1)_15:1
674.5	241.2	PE(31:1)_15:0
674.5	253.2	PE(31:1)_16:1
674.5	255.2	PE(31:1)_16:0
674.5	253.2	PE(O-32:1)_16:1
676.5	241.2	PE(31:0)_15:0
676.5	255.2	PE(31:0)_16:0
676.5	255.2	PE(O-32:0)_16:0
686.5	253.2	PE(32:2)_16:1
688.5	253.2	PE(32:1)_16:1
688.5	255.2	PE(32:1)_16:0
690.5	255.2	PE(32:0)_16:0
696.5	239.2	PE(33:4)_15:1
696.5	277.2	PE(33:4)_18:3
698.5	239.2	PE(33:3)_15:1
698.5	241.2	PE(33:3)_15:0
698.5	277.2	PE(33:3)_18:3
698.5	279.2	PE(33:3)_18:2
698.5	253.2	PE(P-34:2)_16:1
698.5	277.2	PE(O-34:3)_18:3
700.5	239.2	PE(33:2)_15:1
700.5	241.2	PE(33:2)_15:0
700.5	253.2	PE(33:2)_16:1
700.5	267.2	PE(33:2)_17:1
700.5	279.2	PE(33:2)_18:2
700.5	281.2	PE(33:2)_18:1
700.5	253.2	PE(P-34:1)_16:1
700.5	255.2	PE(P-34:1)_16:0
700.5	279.2	PE(O-34:2)_18:2
702.5	239.2	PE(33:1)_15:1
702.5	241.2	PE(33:1)_15:0
702.5	253.2	PE(33:1)_16:1
702.5	255.2	PE(33:1)_16:0
702.5	267.2	PE(33:1)_17:1
702.5	269.2	PE(33:1)_17:0
702.5	281.2	PE(33:1)_18:1

702.5	283.3	PE(33:1)_18:0
702.5	253.2	PE(O-34:1)_16:1
702.5	255.2	PE(P-34:0)_16:0
702.5	281.2	PE(O-34:1)_18:1
704.5	241.2	PE(33:0)_15:0
704.5	255.2	PE(33:0)_16:0
704.5	269.2	PE(33:0)_17:0
704.5	283.3	PE(33:0)_18:0
704.6	255.2	PE(O-34:0)_16:0
704.6	283.3	PE(O-34:0)_18:0
710.5	253.2	PE(34:4)_16:1
710.5	277.2	PE(34:4)_18:3
712.5	253.2	PE(34:3)_16:1
712.5	255.2	PE(34:3)_16:0
712.5	277.2	PE(34:3)_18:3
712.5	279.2	PE(34:3)_18:2
714.5	253.2	PE(34:2)_16:1
714.5	255.2	PE(34:2)_16:0
714.5	279.2	PE(34:2)_18:2
714.5	281.2	PE(34:2)_18:1
716.5	253.2	PE(34:1)_16:1
716.5	255.2	PE(34:1)_16:0
716.5	281.2	PE(34:1)_18:1
716.5	283.3	PE(34:1)_18:0
718.5	255.2	PE(34:0)_16:0
718.5	283.3	PE(34:0)_18:0
720.5	239.2	PE(35:6)_15:1
720.5	301.2	PE(35:6)_20:5
722.5	239.2	PE(35:5)_15:1
722.5	241.2	PE(35:5)_15:0
722.5	301.2	PE(35:5)_20:5
722.5	303.2	PE(35:5)_20:4
722.5	277.2	PE(P-36:4)_18:3
722.5	301.2	PE(O-36:5)_20:5
724.5	239.2	PE(35:4)_15:1
724.5	241.2	PE(35:4)_15:0
724.5	267.2	PE(35:4)_17:1
724.5	277.2	PE(35:4)_18:3
724.5	303.2	PE(35:4)_20:4
724.5	305.2	PE(35:4)_20:3
724.5	277.2	PE(P-36:3)_18:3
724.5	279.2	PE(P-36:3)_18:2
724.5	303.2	PE(O-36:4)_20:4
726.5	241.2	PE(35:3)_15:0
726.5	267.2	PE(35:3)_17:1
726.5	269.2	PE(35:3)_17:0
726.5	277.2	PE(35:3)_18:3

726.5	279.2	PE(35:3)_18:2
726.5	305.2	PE(35:3)_20:3
726.5	277.2	PE(O-36:3)_18:3
726.5	279.2	PE(P-36:2)_18:2
726.5	281.2	PE(P-36:2)_18:1
726.5	305.2	PE(O-36:3)_20:3
728.5	253.2	PE(35:2)_16:1
728.5	267.2	PE(35:2)_17:1
728.5	269.2	PE(35:2)_17:0
728.5	279.2	PE(35:2)_18:2
728.5	281.2	PE(35:2)_18:1
728.5	295.3	PE(35:2)_19:1
728.6	279.2	PE(O-36:2)_18:2
728.6	281.2	PE(P-36:1)_18:1
728.6	283.3	PE(P-36:1)_18:0
730.5	253.2	PE(35:1)_16:1
730.5	255.2	PE(35:1)_16:0
730.5	267.2	PE(35:1)_17:1
730.5	269.2	PE(35:1)_17:0
730.5	281.2	PE(35:1)_18:1
730.5	283.3	PE(35:1)_18:0
730.5	295.3	PE(35:1)_19:1
730.5	297.3	PE(35:1)_19:0
730.6	253.2	PE(O-36:1)_16:1
730.6	281.2	PE(O-36:1)_18:1
730.6	283.3	PE(P-36:0)_18:0
732.6	255.2	PE(35:0)_16:0
732.6	269.2	PE(35:0)_17:0
732.6	283.3	PE(35:0)_18:0
732.6	297.3	PE(35:0)_19:0
732.6	255.2	PE(O-36:0)_16:0
732.6	283.3	PE(O-36:0)_18:0
734.5	253.2	PE(36:6)_16:1
734.5	277.2	PE(36:6)_18:3
734.5	301.2	PE(36:6)_20:5
736.5	253.2	PE(36:5)_16:1
736.5	255.2	PE(36:5)_16:0
736.5	277.2	PE(36:5)_18:3
736.5	279.2	PE(36:5)_18:2
736.5	301.2	PE(36:5)_20:5
736.5	303.2	PE(36:5)_20:4
738.5	253.2	PE(36:4)_16:1
738.5	255.2	PE(36:4)_16:0
738.5	277.2	PE(36:4)_18:3
738.5	279.2	PE(36:4)_18:2
738.5	281.2	PE(36:4)_18:1
738.5	303.2	PE(36:4)_20:4

738.5	305.2	PE(36:4)_20:3
740.5	255.2	PE(36:3)_16:0
740.5	277.2	PE(36:3)_18:3
740.5	279.2	PE(36:3)_18:2
740.5	281.2	PE(36:3)_18:1
740.5	283.3	PE(36:3)_18:0
740.5	305.2	PE(36:3)_20:3
742.5	279.2	PE(36:2)_18:2
742.5	281.2	PE(36:2)_18:1
742.5	283.3	PE(36:2)_18:0
744.6	281.2	PE(36:1)_18:1
744.6	283.3	PE(36:1)_18:0
746.5	301.2	PE(P-38:6)_20:5
746.6	283.3	PE(36:0)_18:0
748.5	267.2	PE(37:6)_17:1
748.5	301.2	PE(37:6)_20:5
748.5	301.2	PE(P-38:5)_20:5
748.5	303.2	PE(P-38:5)_20:4
750.5	267.2	PE(37:5)_17:1
750.5	269.2	PE(37:5)_17:0
750.5	301.2	PE(37:5)_20:5
750.5	303.2	PE(37:5)_20:4
750.5	301.2	PE(O-38:5)_20:5
750.5	303.2	PE(P-38:4)_20:4
750.5	305.2	PE(P-38:4)_20:3
752.5	267.2	PE(37:4)_17:1
752.5	269.2	PE(37:4)_17:0
752.5	277.2	PE(37:4)_18:3
752.5	295.3	PE(37:4)_19:1
752.5	303.2	PE(37:4)_20:4
752.5	305.2	PE(37:4)_20:3
752.6	303.2	PE(O-38:4)_20:4
752.6	305.2	PE(P-38:3)_20:3
754.5	269.2	PE(37:3)_17:0
754.5	277.2	PE(37:3)_18:3
754.5	279.2	PE(37:3)_18:2
754.5	295.3	PE(37:3)_19:1
754.5	297.3	PE(37:3)_19:0
754.5	305.2	PE(37:3)_20:3
754.6	277.2	PE(O-38:3)_18:3
754.6	305.2	PE(O-38:3)_20:3
756.6	279.2	PE(37:2)_18:2
756.6	281.2	PE(37:2)_18:1
756.6	295.3	PE(37:2)_19:1
756.6	297.3	PE(37:2)_19:0
756.6	279.2	PE(O-38:2)_18:2
758.5	277.2	PE(38:8)_18:3

758.5	301.2	PE(38:8)_20:5
758.6	281.2	PE(37:1)_18:1
758.6	283.3	PE(37:1)_18:0
758.6	295.3	PE(37:1)_19:1
758.6	297.3	PE(37:1)_19:0
758.6	281.2	PE(O-38:1)_18:1
760.5	277.2	PE(38:7)_18:3
760.5	279.2	PE(38:7)_18:2
760.5	301.2	PE(38:7)_20:5
760.5	303.2	PE(38:7)_20:4
760.6	283.3	PE(37:0)_18:0
760.6	297.3	PE(37:0)_19:0
760.6	283.3	PE(O-38:0)_18:0
762.5	277.2	PE(38:6)_18:3
762.5	279.2	PE(38:6)_18:2
762.5	281.2	PE(38:6)_18:1
762.5	301.2	PE(38:6)_20:5
762.5	303.2	PE(38:6)_20:4
762.5	305.2	PE(38:6)_20:3
764.5	279.2	PE(38:5)_18:2
764.5	281.2	PE(38:5)_18:1
764.5	283.3	PE(38:5)_18:0
764.5	301.2	PE(38:5)_20:5
764.5	303.2	PE(38:5)_20:4
764.5	305.2	PE(38:5)_20:3
766.5	281.2	PE(38:4)_18:1
766.5	283.3	PE(38:4)_18:0
766.5	303.2	PE(38:4)_20:4
766.5	305.2	PE(38:4)_20:3
768.6	283.3	PE(38:3)_18:0
768.6	305.2	PE(38:3)_20:3
776.5	295.3	PE(39:6)_19:1
776.5	301.2	PE(39:6)_20:5
778.5	295.3	PE(39:5)_19:1
778.5	297.3	PE(39:5)_19:0
778.5	301.2	PE(39:5)_20:5
778.5	303.2	PE(39:5)_20:4
778.6	301.2	PE(O-40:5)_20:5
780.6	295.3	PE(39:4)_19:1
780.6	297.3	PE(39:4)_19:0
780.6	303.2	PE(39:4)_20:4
780.6	305.2	PE(39:4)_20:3
780.6	303.2	PE(O-40:4)_20:4
782.5	301.2	PE(40:10)_20:5
782.6	297.3	PE(39:3)_19:0
782.6	305.2	PE(39:3)_20:3
782.6	305.2	PE(O-40:3)_20:3

784.5	301.2	PE(40:9)_20:5
784.5	303.2	PE(40:9)_20:4
786.5	301.2	PE(40:8)_20:5
786.5	303.2	PE(40:8)_20:4
786.5	305.2	PE(40:8)_20:3
788.5	303.2	PE(40:7)_20:4
788.5	305.2	PE(40:7)_20:3
790.5	305.2	PE(40:6)_20:3
