# Supplementary material for: A Caenorhabditis elegans model for ether lipid biosynthesis and function
Source: J Lipid Res. 2016 Feb;57(2):265–75. doi: 10.1194/jlr.M064808 (PMC4727422; doi:10.1194/jlr.M064808)
Supplement: Supplemental Data [file 10.1194_M064808_jlr.M064808-3.pdf]

Table S2. Single reaction monitoring (SRM) transitions from $[M+H]^+$ molecular ions to plasmalogen-specific fragments, which are diagnostic for *sn*-1 and *sn*-2 radyl chains (20). Transition name includes the lipid class phosphatidylethanolamine (PE), followed by the targeted apparent species in brackets. "P-" indicates apparent 1-O-Alk-1'-enyl-2-acyl-PE species. The vinyl ether double bond of 1-O-Alk-1'-enyl-2-acyl-PE species was taken into account for the apparent species notation.

Precursor ion *m*/*z*	Fragment ion *m*/*z*	Transition name
700.5	390.3	PE(P-34:3)_sn-1
700.5	311.3	PE(P-34:3)_sn-2
702.5	390.3	PE(P-34:2)_sn-1
702.5	392.3	PE(P-34:2)_sn-1
702.5	313.3	PE(P-34:2)_sn-2
702.5	311.3	PE(P-34:2)_sn-2
704.6	392.3	PE(P-34:1)_sn-1
704.6	313.3	PE(P-34:1)_sn-2
724.5	390.3	PE(P-36:5)_sn-1
724.5	335.3	PE(P-36:5)_sn-2
726.5	390.3	PE(P-36:4)_sn-1
726.5	392.3	PE(P-36:4)_sn-1
726.5	337.3	PE(P-36:4)_sn-2
726.5	335.3	PE(P-36:4)_sn-2
728.6	390.3	PE(P-36:3)_sn-1
728.6	392.3	PE(P-36:3)_sn-1
728.6	339.3	PE(P-36:3)_sn-2
728.6	337.3	PE(P-36:3)_sn-2
730.6	390.3	PE(P-36:2)_sn-1
730.6	392.3	PE(P-36:2)_sn-1
730.6	341.3	PE(P-36:2)_sn-2
730.6	339.3	PE(P-36:2)_sn-2
732.6	392.3	PE(P-36:1)_sn-1
732.6	341.3	PE(P-36:1)_sn-2
748.5	390.3	PE(P-38:7)_sn-1
748.5	359.3	PE(P-38:7)_sn-2
750.5	390.3	PE(P-38:6)_sn-1
750.5	392.3	PE(P-38:6)_sn-1
750.5	361.3	PE(P-38:6)_sn-2
750.5	359.3	PE(P-38:6)_sn-2
752.6	390.3	PE(P-38:5)_sn-1
752.6	392.3	PE(P-38:5)_sn-1
752.6	363.3	PE(P-38:5)_sn-2
752.6	361.3	PE(P-38:5)_sn-2
754.6	392.3	PE(P-38:4)_sn-1
754.6	363.3	PE(P-38:4)_sn-2
