# Supplementary material for: A Caenorhabditis elegans model for ether lipid biosynthesis and function
Source: J Lipid Res. 2016 Feb;57(2):265–75. doi: 10.1194/jlr.M064808 (PMC4727422; doi:10.1194/jlr.M064808)
Supplement: Supplemental Data [file 10.1194_M064808_jlr.M064808-4.pdf]

Table S3. Identity confirmation of 17 phosphatidylethanolamine ether lipids by untargeted ultra performance liquid chromatography time-of-flight (UPLC-TOF) analysis.

Phophatidylethanolamine molecular species	Exact *m/z* (calculated)	Accurate *m/z* (measured)	Absolute *m/z* error [mDa]	Relative *m/z* error [ppm]
O-16:0/18:1	702.5443	702.5450	0.7	1.0
O-16:0/18:2	700.5287	700.5270	1.7	2.4
O-16:0/20:3	726.5443	726.5483	4.0	5.5
O-18:0/16:0	704.5600	704.5598	0.2	0.2
O-18:0/16:1	702.5443	702.5450	0.7	1.0
O-18:0/18:0	732.5913	732.5927	1.4	2.0
O-18:0/18:1	730.5756	730.5756	0.0	0.0
O-18:0/18:2	728.5600	728.5626	2.6	3.6
O-18:0/18:3	726.5443	726.5483	4.0	5.5
O-18:0/20:3	754.5756	754.5717	3.9	5.2
O-18:0/20:4	752.5600	752.5601	0.1	0.2
O-20:0/18:1	758.6069	758.6093	2.4	3.1
O-20:0/18:2	756.5913	756.5904	0.9	1.1
P-18:0/18:1	728.5600	728.5626	2.6	3.6
P-18:0/18:2	726.5443	726.5406	3.7	5.1
P-18:0/20:3	752.5600	752.5601	0.1	0.2
P-18:0/20:5	748.5287	748.5259	2.8	3.7
