# Supplementary material for: A Caenorhabditis elegans model for ether lipid biosynthesis and function
Source: J Lipid Res. 2016 Feb;57(2):265–75. doi: 10.1194/jlr.M064808 (PMC4727422; doi:10.1194/jlr.M064808)
Supplement: Supplemental Data [file 10.1194_M064808_jlr.M064808-5.pdf]

**Table S4. Primers used for quantitative real-time RT-PCR.**

| Gene | Forward Primer | Reverse Primer |
|---|---|---|
| | | |
| cdc-42 | CATTCGAGAATGTCCGAGAA | CTGCTTTCAATTCCTTTGCC |
| fat-5 | CGATTTGTACGAGGATCCGGTG | CAGTGGGAGACACTGTTGATGC |
| fat-6 | TCTACCAGCTCATCTTCGAGGC | GATCACGAGCCCATTCGATGAC |
| fat-7 | GGAAGGAGACAGCATTCATTGCG | GTCTTGTGGGAATGTGTGGTGG |
| elo-2 | ACTTGCTCTCTGGAACTTCGGG | GCTTTCGACATCACAAAGGCCC |
| pod-2 | AGAAGTGCCGAGAACACCTT | ACGGAGACTTGAGCACACTG |
