# Supplementary material for: A Caenorhabditis elegans model for ether lipid biosynthesis and function
Source: J Lipid Res. 2016 Feb;57(2):265–75. doi: 10.1194/jlr.M064808 (PMC4727422; doi:10.1194/jlr.M064808)
Supplement: Supplemental Data [file 10.1194_M064808_jlr.M064808-6.pdf]

Table S5. Lifespan analysis summary

Strain	Mean lifespan (days)	% different from N2	P-value	# subjects	# deaths	censored
N2-1	16.37			97	74	23
*N2-2	16.77			90	75	15
N2-3	18.18			75	60	13
fard-1 (wa28)-1	12.94	-21.0	<0.0001	115	107	8
*fard-1 (wa28)-2	11.90	-29.0	<0.0001	91	85	6
fard-1 (wa2)-3	11.43	-37.1	<0.0001	99	78	21
ads-1 (wa3)-1	11.63	-29.0	<0.0001	112	95	17
*ads-1 (wa3)-2	11.80	-29.6	<0.0001	111	97	14
ads-1 (wa3)-3	12.49	-31.2	<0.0001	75	60	15
acl-7 (wa20)-1	12.45	-23.9	<0.0001	92	68	24
*acl-7(wa20)-2	11.72	-30.1	<0.0001	99	80	19
acl-7(wa20)-3	10.35	-43.1	<0.0001	86	70	16

*Used for Fig 5C
