# Supplementary material for: A Caenorhabditis elegans model for ether lipid biosynthesis and function
Source: J Lipid Res. 2016 Feb;57(2):265–75. doi: 10.1194/jlr.M064808 (PMC4727422; doi:10.1194/jlr.M064808)
Supplement: Supplemental Data [file 10.1194_M064808_jlr.M064808-7.pdf]

Table S6. Fatty acid composition of ether lipid-deficient mutant strains and RNAi treated strains as determined by GC-MS.

	14:0	15iso	16:0	17iso	16:1	18:0DMA	17d	18:0	18:1n-9	18:1n-7	18:2	19d	20:0-18:3	20:3	20:4	20:4n-3	20:5
Mutants																	
**N2 ave**	**1.23%**	**1.79%**	**3.52%**	**3.63%**	**1.04%**	**3.47%**	**13.91%**	**7.30%**	**1.90%**	**11.21%**	**5.71%**	**9.81%**	**0.63%**	**4.12%**	**1.64%**	**7.82%**	**21.28%**
N2 St Dev	0.31%	0.08%	0.68%	0.39%	0.27%	0.68%	4.59%	1.11%	0.31%	0.65%	1.26%	1.19%	0.39%	0.15%	0.20%	1.83%	2.64%
**fard-1(wa2) ave**	**1.43%**	**2.96%**	**3.39%**	**2.46%**	**1.10%**	**0.38%**	**13.07%**	**12.90%**	**1.58%**	**11.61%**	**5.60%**	**8.41%**	**3.07%**	**3.26%**	**1.50%**	**4.73%**	**22.54%**
fard-1(wa2) St Dev	0.16%	0.52%	0.10%	0.18%	0.09%	0.02%	3.15%	0.92%	0.21%	0.47%	0.44%	1.63%	0.23%	0.24%	0.23%	0.68%	3.67%
**fard-1(wa28) ave**	**0.72%**	**1.88%**	**2.12%**	**3.40%**	**0.58%**	**0.00%**	**10.24%**	**16.77%**	**1.21%**	**9.92%**	**5.64%**	**9.81%**	**1.73%**	**3.69%**	**2.11%**	**5.24%**	**24.41%**
fard-1(wa28) St Dev	0.08%	0.35%	0.26%	0.26%	0.11%	0.00%	1.80%	1.50%	0.10%	1.34%	0.50%	1.14%	0.59%	0.46%	0.14%	0.89%	2.04%
**ads-1(wa3) ave**	**1.10%**	**2.23%**	**2.86%**	**2.43%**	**0.90%**	**0.00%**	**8.68%**	**16.28%**	**1.22%**	**10.20%**	**6.08%**	**7.11%**	**3.49%**	**3.68%**	**2.36%**	**4.81%**	**25.88%**
ads-1(wa3) St Dev	0.06%	0.31%	0.05%	0.11%	0.21%	0.00%	1.30%	0.68%	0.12%	1.16%	0.39%	0.63%	0.18%	0.16%	0.18%	0.28%	2.07%
**acl-7(wa20) ave**	**0.00%**	**2.06%**	**2.39%**	**3.46%**	**0.58%**	**0.34%**	**15.60%**	**15.38%**	**1.72%**	**6.75%**	**5.04%**	**11.92%**	**2.68%**	**5.05%**	**1.86%**	**6.21%**	**18.97%**
acl-7(wa20) St Dev	0.00%	0.26%	0.71%	0.31%	0.16%	0.02%	0.89%	0.10%	0.38%	0.66%	0.76%	0.69%	0.70%	0.10%	0.08%	0.93%	2.45%
RNAi (N2 worms)																	
**Empty vector ave**	**1.15%**	**1.65%**	**4.27%**	**2.39%**	**4.50%**	**2.80%**	**7.05%**	**5.52%**	**1.93%**	**17.34%**	**5.26%**	**1.70%**	**2.60%**	**3.82%**	**2.49%**	**7.33%**	**28.20%**
Empty vector St Dev	0.00%	0.13%	0.23%	0.04%	0.05%	0.09%	0.11%	0.10%	0.16%	0.18%	0.04%	0.08%	0.08%	0.23%	0.02%	0.14%	0.26%
**fard-1(RNAi) ave**	**1.32%**	**1.95%**	**3.65%**	**2.13%**	**5.53%**	**0.68%**	**6.12%**	**12.49%**	**1.62%**	**15.27%**	**3.48%**	**1.45%**	**3.39%**	**4.01%**	**2.91%**	**6.49%**	**27.51%**
fard-1(RNAi) St Dev	0.06%	0.34%	0.15%	0.06%	0.13%	0.01%	0.34%	0.06%	0.11%	0.11%	0.05%	0.01%	0.06%	0.17%	0.05%	0.10%	0.45%
**ads-1(RNAi) ave**	**1.35%**	**2.04%**	**3.65%**	**2.16%**	**4.87%**	**1.40%**	**6.86%**	**10.28%**	**1.35%**	**16.14%**	**3.36%**	**1.87%**	**3.18%**	**3.96%**	**2.71%**	**6.91%**	**27.92%**
ads-1(RNAi) St Dev	0.09%	0.29%	0.17%	0.15%	0.49%	0.17%	1.44%	0.56%	0.03%	0.21%	0.18%	0.20%	0.09%	0.43%	0.13%	0.26%	0.97%
**acl-7(RNAi) ave**	**1.39%**	**2.16%**	**3.70%**	**2.06%**	**5.80%**	**1.26%**	**6.38%**	**10.81%**	**1.54%**	**15.25%**	**3.41%**	**1.55%**	**3.07%**	**3.97%**	**2.76%**	**6.93%**	**27.97%**
acl-7(RNAi) St Dev	0.13%	0.43%	0.03%	0.07%	0.28%	0.07%	0.82%	0.31%	0.03%	0.06%	0.12%	0.13%	0.11%	0.15%	0.21%	0.21%	1.10%
